# Associations Between Kidney Function and Risk of Cognitive Impairment Among Chinese Older Adults

**DOI:** 10.1111/ggi.70859

**Published:** 2026-09-27

**Authors:** Xin Wang, Yue Zhang, Zhigang Wang, Xiaolei Liu

**Affiliations:** ^1^ Department of Neurology, Shanxi Bethune Hospital Third Hospital of Shanxi Medical University, Shanxi Academy of Medical Sciences, Tongji Shanxi Hospital Taiyuan China; ^2^ Department of Epidemiology, School of Public Health Shanxi Medical University, Key Laboratory of Coal Environmental Pathogenicity and Prevention, Ministry Education Taiyuan China; ^3^ Clinical Research Center for Neurological Diseases of Guangxi Province Guilin Medical University Guilin China; ^4^ Key Laboratory of Environmental Exposomics and Entire Lifecycle Health of Guangxi Province Guilin Medical University Guilin China; ^5^ Key Laboratory of Brain and Cognition of Guangxi Province Guilin Medical University Guilin China; ^6^ Key Laboratory of Diabetic Systems Medicine of Guangxi Province Guilin Medical University Guilin China

**Keywords:** CLHLS, cognitive impairment, kidney function, older adults, risk

## Abstract

**Background:**

The association between kidney function and dementia remains inconsistent across studies, and its relationship in older adults is unclear. This study aimed to investigate the longitudinal associations between kidney dysfunction, assessed by estimated glomerular filtration rate (eGFR) and albuminuria, and cognitive impairment (CI) in a national cohort of Chinese older adults.

**Methods:**

We included individuals aged ≥ 65 years from the 2011 and 2014 waves of the Chinese Longitudinal Healthy Longevity Survey (CLHLS). Cognitive function was assessed using the Chinese version of the Mini‐Mental State Examination (MMSE). Kidney function was evaluated by eGFR (CKD‐EPI equation) and the urinary albumin‐to‐creatinine ratio (ACR), classified by KDIGO categories and quartiles. Multivariable logistic regression models were used to assess cross‐sectional (*n* = 3364) and prospective (*n* = 1195) associations, controlling for sociodemographic and clinical confounders. The false discovery rate was controlled for multiple comparisons.

**Results:**

In cross‐sectional analysis, both lower eGFR and higher ACR were independently associated with higher odds of prevalent CI. Prospectively, over a 3‐year follow‐up, 143 of 1195 cognitively normal participants developed CI. Compared to those with normal kidney function, participants with impaired (eGFR < 60 mL/min/1.73 m^2^) kidney function had a significantly higher risk of incident CI (OR = 1.744, 95% CI: 1.162–2.616). Similarly, elevated ACR (categories A2 and A3) was associated with increased incident CI risk (A2: OR = 1.801, 95% CI: 1.111–2.920; A3: OR = 2.650, 95% CI: 1.541–4.557).

**Conclusions:**

Impaired kidney function, indicated by reduced eGFR and elevated ACR, is independently associated with an increased risk of cognitive impairment in older adults. These findings support integrating cognitive screening into the clinical management of elderly patients with chronic kidney disease.

## Graphical abstract

This graphical abstract illustrates the association between kidney function and risk of cognitive impairment among Chinese older adults from the CLHLS cohort. Reduced eGFR and elevated ACR assessed at baseline were significantly associated with higher odds of both prevalent and incident cognitive impairment, after adjusting for sociodemographic and clinical confounders. The proposed mechanisms include decreased renal clearance of Alzheimer's disease‐related proteins (Aβ and tau), microvascular endothelial injury, neuroinflammation, and oxidative stress. These findings support the integration of cognitive screening into the clinical management of chronic kidney disease in elderly populations.

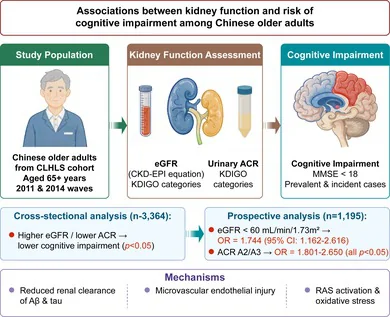

## 1 Introduction

Dementia, a chronic and progressive clinical syndrome characterized by the deterioration of multiple cognitive domains, presents a growing global health challenge. According to the Global Burden of Disease Study, the worldwide prevalence of dementia was estimated at 57.4 million individuals in 2019, with projections indicating a rise to 152.8 million by 2050 [1]. In China, approximately 15.07 million people aged 60 years and above were living with dementia [2]. The rising prevalence of dementia imposes a substantial burden on individuals, families, and society as a whole. Despite this, effective treatments for dementia remain limited, underscoring the urgent need to identify modifiable risk factors for disease prevention.

Alzheimer's disease (AD), the most common form of dementia, is pathologically characterized by the abnormal accumulation of amyloid‐beta (Aβ) and hyperphosphorylated tau (p‐tau) proteins. Prior research indicated that these pathological proteins can be transported from the central nervous system into peripheral biofluids via the blood–brain barrier (BBB) and the glymphatic system [3]. As a vital metabolic organ, the kidney plays a vital role in metabolite excretion and systemic homeostasis maintenance. Both in vitro and in vivo trials have confirmed that the kidney is responsible for the peripheral clearance of Aβ and tau from the blood [4, 5]. Growing evidence links kidney function (KF) to dementia risk and its underlying pathological processes. For instance, a community‐based prospective cohort study reported elevated albuminuria was associated with an increased risk of dementia [6]. Similarly, a large‐scale analysis of 202 702 participants aged 60 years and older from the UK biobank demonstrated that individuals with impaired KF had a 1.42‐fold higher risk of developing dementia compared to those with normal KF [7]. Another prospective cohort study involving 3033 patients with Stage 3–4 chronic kidney disease (CKD) demonstrated that lower estimated glomerular filtration rate (eGFR) was correlated with poorer cognitive performance and a higher incidence of cognitive impairment [8]. More recent studies have further associated impaired KF with brain structural changes, including total brain volume atrophy and medial temporal lobe atrophy [9, 10]. A community‐based cohort study also showed that reduced KF was strongly linked to a greater burden of cerebral amyloid angiopathy [11]. Additionally, prior studies have documented inverse correlations between eGFR and peripheral Aβ levels and tau levels [5, 12].

Despite these findings, the association between KF and dementia remains inconsistent across studies. For example, a 17‐year longitudinal study by Hannah et al. found no significant association between baseline impaired KF and increased dementia risk [13]. Likewise, Mendelian randomization analyses by Kjaergaard et al. observed no correlation between eGFR and dementia risk [14], and Helmer et al. also failed to identify a link between low eGFR levels and cognitive impairment [15]. These discrepancies may stem from inadequate adjustment for confounding variables, differences in study populations, and variations in the methods used to assess KF and cognitive function. Furthermore, the relationship between KF and dementia in the elderly—a population with a rapidly growing incidence and substantially higher prevalence of dementia compared to younger adults [16] remains poorly understood. Most existing evidence is derived from Western populations, leaving a gap in data specific to Chinese older adults. Furthermore, few studies have comprehensively evaluated KF using both eGFR and the albumin‐to‐creatinine ratio (ACR) in relation to dementia risk, limiting the robustness of existing conclusions. Addressing these research gaps is crucial for developing targeted dementia prevention strategies for the Chinese elderly, a group with one of the largest dementia burdens globally. Accurately determining whether reduced eGFR is an independent risk factor for cognitive decline in older adults is therefore critical; this would not only clarify the pathogenic role of impaired KF in cognitive impairment but also inform more targeted strategies for dementia risk prediction and prevention in this high‐risk population.

Therefore, in this present study, we performed cross‐sectional and longitudinal analyses using data from the Chinese Longitudinal Healthy Longevity Survey (CLHLS) cohort to investigate the associations between KF—as assessed by eGFR and ACR—and the risk of all‐cause dementia (ACD) in older adults in China.

## 2 Material and Methods

### 2.1 Study Participants

Data from the 2011 and 2014 waves of the CLHLS were used in this study to assess the association between KF and the risk of ACD. The CLHLS is an ongoing, prospective cohort study designed to investigate the health status of older adults across 23 provinces in China. This survey was initiated in 1998 and has been conducted biennially or triennially, with detailed protocols described previously [17]. The data are publicly available through the Peking University Open Research Data Platform (https://opendata.pku.edu.cn/dataverse/CHADS).

In the 2011 and 2014 waves, 2317 and 2499 participants aged 60 years or older, respectively, had available blood creatinine data. After excluding those with missing Mini‐Mental State Examination (MMSE) scores (60 in the 2011 wave and 27 in the 2014 wave), a total of 2257 individuals from the 2011 wave and 2472 from the 2014 wave were included. Among the 2472 participants in the 2014 wave, 1365 were followed up from the 2011 wave, while 1107 were newly recruited in the 2014 wave. For cross‐sectional analyses, we combined the 2257 participants from the 2011 wave and 1107 new participants from the 2014 wave. For prospective analyses, we included 1195 participants from the follow‐up cohort who had normal cognitive function at baseline. A flowchart detailing participant screening is provided in Figure 1.

**FIGURE 1:**
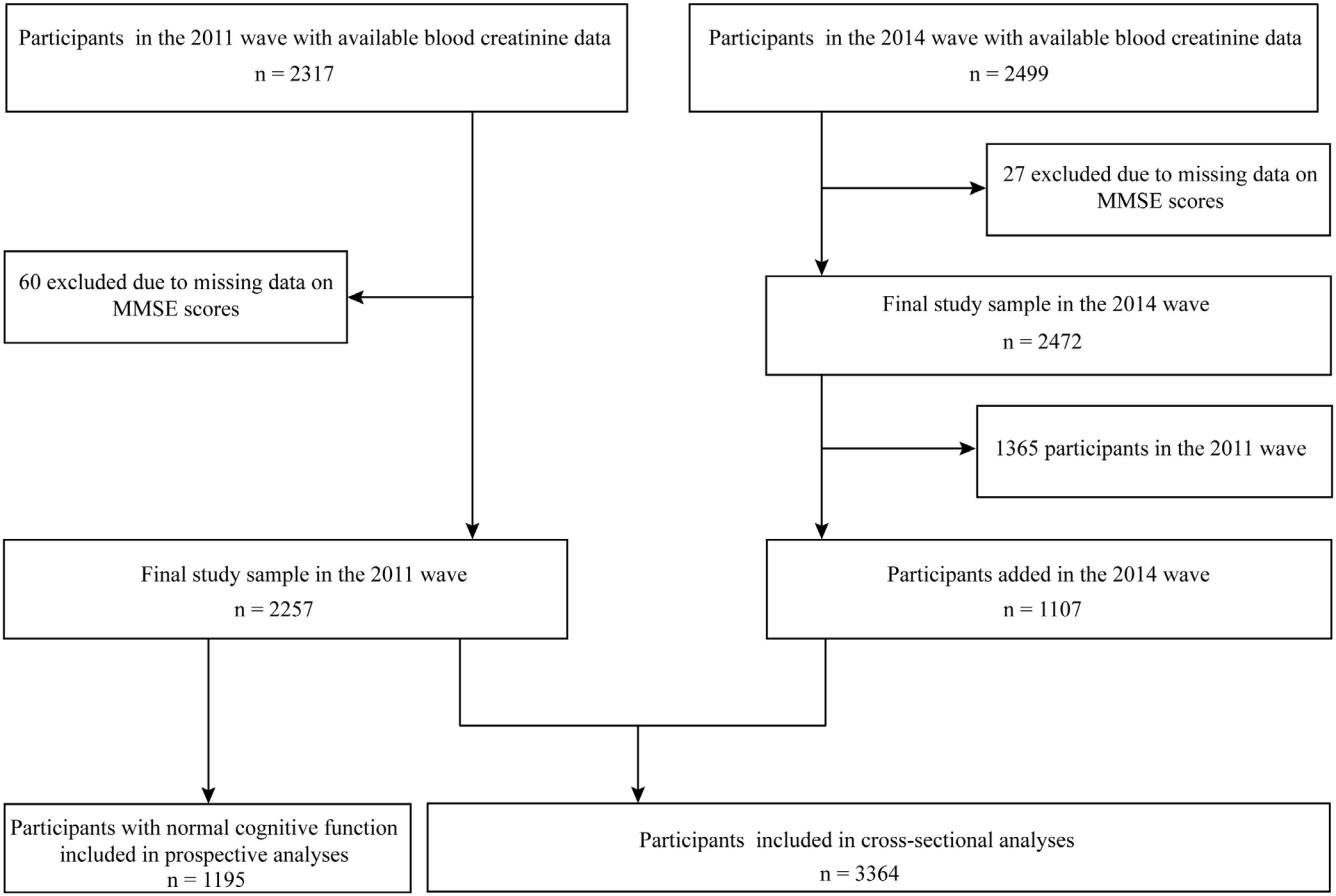
Detailed flowchart of participant selection. MMSE, Mini‐Mental State Examination.

The CLHLS study was approved by the Ethics Committee of Peking University (IRB00001052‐13074) and conducted in accordance with the Declaration of Helsinki. Written informed consent was obtained from all participants or their legal representatives.

### 2.2 Covariates

Trained professionals collected data through face‐to‐face interviews using structured questionnaires. Covariates included age, sex, ethnicity (Han vs. others), marital status (married, widowed, or other), years of education, smoking history (≥ 20 pack‐years), alcohol consumption (≥ twice weekly for over 1 year), regular physical activity (defined as intentional exercise at least once per week, such as walking, jogging, qigong, tai chi, or square dancing), body mass index (BMI), and history of physician‐diagnosed diseases including hypertension, diabetes, stroke, cardiovascular disease, respiratory disorders, and malignant tumors.

### 2.3 Cognitive Function Assessment

Cognitive function was assessed using the Chinese version of the MMSE, which ranges from 0 to 30 points, with higher scores indicating better cognitive performance [18]. All items were completed by the participants themselves without proxy assistance. In line with previous studies [19, 20], participants were classified as having cognitive impairment (CI) if their MMSE score was below 18.

### 2.4 KF Measurement

Fasting venous blood samples and first‐morning midstream urine specimens were collected in the morning and immediately transported to a local hospital for analysis. Serum creatinine and urinary ACR were measured on the same day. KF was evaluated using eGFR and urinary ACR. The eGFR was calculated using the Chronic Kidney Disease Epidemiology Collaboration (CKD‐EPI) equation based on serum creatinine [21]. This equation is preferred in older populations and individuals with relatively preserved KF due to its higher accuracy and better predictive ability for vascular events and end‐stage kidney disease [22]. Both eGFR and ACR were classified according to the Improving Global Outcomes (KDIGO) guidelines [23]. To ensure adequate statistical power across exposure groups, eGFR was categorized into three clinically relevant groups aligned with KDIGO 2024 guidance: normal (≥ 60 mL/min/1.73 m^2^), low (60 > eGFR ≥ 45 mL/min/1.73 m^2^), and poor (< 45 mL/min/1.73 m^2^) KF, as indicated in previous literature [24]. Urinary ACR was classified into KDIGO categories A1–A3: A1 (< 3 mg/mmol), A2 (3–30 mg/mmol), and A3 (> 30 mg/mmol).

### 2.5 Statistical Analyses

Continuous variables were compared using Student's *t*‐test or Mann–Whitney *U* test, as appropriate, while categorical variables were analyzed using the Chi‐squared test. Due to skewed distributions, eGFR and ACR were presented as median with interquartile range. Binary logistic regression was used to estimate odds ratios (ORs) and 95% confidence intervals (CIs) for the associations of eGFR and ACR with CI risk in both cross‐sectional and prospective analyses. Models were sequentially adjusted for confounders: Model 1 (unadjusted); Model 2 (Model 1 + age, sex, ethnicity, marital status, and education); Model 3 (Model 2 + smoking, drinking status and regular physical activity); Model 4 (Model 3 + BMI, medical history of hypertension, diabetes and stroke). The false discovery rate (FDR) for multiple comparisons across exposure categories was controlled using the Benjamini‐Hochberg procedure. Both eGFR and ACR were classified using two approaches: according to the KDIGO guidelines and based on quartiles. To explore potential nonlinear relationships, restricted cubic spline regression was further applied in both the cross‐sectional and prospective analysis. All statistical analyses were performed using SPSS version 26.0 (IBM Corp., Armonk, NY, USA), and a two‐tailed *p* < 0.05 was considered statistically significant.

## 3 Results

### 3.1 Study Participants

In the cross‐sectional study of combined samples, a total of 3364 participants were included, with a mean age of 86.16 ± 11.85 years. Among them, 2667 were cognitively normal (CN) and 697 had CI. No significant differences were observed between the two groups regarding ethnicity, BMI, or history of hypertension, cardiovascular disease, respiratory disorders, or cancer. Compared to the CN group, individuals with CI were significantly older, more likely to be female and widowed, had fewer years of schooling, and exhibited higher proportions of smoking and alcohol consumption, lower rates of regular physical activity, and higher prevalence of diabetes and stroke. In addition, the CI group had significantly lower eGFR and higher ACR levels than the CN group. Detailed demographic and clinical characteristics of the study population are presented in Table 1.

**TABLE 1:** The detailed demographic and clinical characteristics of all participants.

Characteristics	Total (*N* = 3364)	CN (*N* = 2667)	CI (*N* = 697)	*p*	*t*, *χ* ^2^, or *U*	*η* ^2^, *V*, or *r*
Age (years), mean (SD)	86.16 (11.85)	84.55 (11.24)	92.31 (12.14)	**< 0.001**	553 214	0.284
Men (vs women), *n* (%)	1506 (44.77)	1350 (50.62)	156 (22.38)	**< 0.001**	178.188	0.230
Ethnicity (Han vs others), *n* (%)	2997 (89.09)	2379 (89.20)	618 (88.67)	0.686	0.163	0.007
Marital status, *n* (%)				**< 0.001**	221.327	0.256
Married	1265 (37.60)	1169 (43.83)	96 (13.77)			
Widowed	1978 (58.80)	1399 (52.46)	579 (83.07)			
Other	121 (3.60)	99 (3.71)	22 (3.16)			
Years of schooling, mean [M (P25, P75)]	0 (0, 0)	0 (0, 5)	0 (0, 0)	**< 0.001**	625 805	0.229
Smoking history, *n* (%)	835 (24.82)	640 (24.00)	195 (27.98)	**0.030**	4.691	0.037
Drinking history, *n* (%)	699 (20.78)	531 (19.91)	168 (24.10)	**0.015**	5.903	0.042
Regular physical activity, *n* (%)	586 (17.42)	501 (18.79)	85 (12.20)	**< 0.001**	16.682	0.070
BMI (kg/m^2^)	21.21 (3.14)	21.24 (3.15)	21.07 (3.07)	0.213	1.246	0.001
Hypertension, *n* (%)	886 (26.34)	708 (26.55)	178 (25.54)	0.590	0.290	0.009
Diabetes, *n* (%)	251 (7.46)	179 (6.71)	72 (10.33)	**0.001**	10.478	0.056
Stroke, *n* (%)	244 (7.25)	172 (6.45)	72 (10.33)	**< 0.001**	12.371	0.061
Cardiovascular disease, *n* (%)	257 (7.64)	209 (7.84)	48 (6.89)	0.401	0.707	0.015
Respiratory diseases, *n* (%)	282 (8.38)	227 (8.51)	55 (7.89)	0.599	0.277	0.009
Cancer, *n* (%)	14 (0.42)	12 (0.45)	2 (0.29)	0.748	0.354	0.010
eGFR [M (P25, P75)]	74.09 (58.98, 85.50)	75.97 (60.75, 87.17)	65.83 (51.91, 78.23)	**< 0.001**	12.04	0.041
ACR (mg/mmol)^a^	7.40 (2.06, 24.24)	6.29 (1.78, 19.78)	15.88 (4.58, 48.32)	**< 0.001**	519 951	0.298

*Note:* Significant results in bold.

Abbreviations: BMI, body mass index; M, median; P25, 25% quartile, P75, 75% quartile; SD, standard deviation.

^a^ Of all 3364 participants enrolled in the cross‐sectional analysis, 3053 enrolled participants had available ACR values. Among them, 2473 were cognitively normal and 580 were cognitively impaired.

### 3.2 Cross‐Sectional Association Between KF and CI Risk

After adjusting for all relevant confounders, participants in the highest two eGFR quartiles exhibited significantly lower odds of prevalent CI compared to those in the lowest quartile (OR = 0.676, 95% CI: 0.528–0.866 for the third quartile, and OR = 0.530, 95% CI: 0.368–0.763 for the fourth quartile). When eGFR was categorized according to KDIGO guidelines, individuals with impaired and poor KF showed elevated odds of prevalent CI relative to those with normal KF (OR = 1.251, 95% CI: 1.021–1.534 for impaired function; OR = 1.441, 95% CI: 1.093–1.900 for poor function). Regarding ACR, participants in the highest quartile had significantly higher odds of prevalent CI compared to those in the lowest quartile (OR = 2.007, 95% CI: 1.464–2.751). When ACR was classified into KDIGO categories A1–A3, participants in the A2 and A3 groups demonstrated an increased odds of prevalent CI relative to the A1 group (A2: OR = 1.377, 95% CI: 1.045–1.814; A3: OR = 2.272, 95% CI: 1.683–3.067) (Table 2). Restricted cubic spline regression revealed nonlinear associations between eGFR, ACR, and CI prevalence (*p* for nonlinear = 0.016 and 0.001, respectively). The odds of prevalent CI declined progressively with increasing eGFR, with a marked decrease observed when eGFR exceeded 61.55 mL/min/1.73 m^2^. Conversely, the odds of prevalent CI increased with rising ACR, and the increase became more gradual beyond an ACR of 61.98 mg/mmol (shown in Figure 2A,B). To examine whether the association between eGFR and cognitive impairment differed by sex, we included an eGFR × sex interaction term in the fully adjusted logistic regression model. The likelihood ratio test was significant (*χ* ^2^ = 9.29, *p* = 0.002), indicating effect modification by sex. In sex‐stratified analyses, lower eGFR was significantly associated with cognitive impairment in men (OR per mL/min/1.73 m^2^ = 0.975; 95% CI: 0.964–0.986; *p* < 0.001) but not in women (OR = 0.995; 95% CI: 0.988–1.00; *p* = 0.109). The eGFR category × sex interaction was also significant (*p* = 0.024). Predicted probability curves are shown in Figure S1.

**TABLE 2:** Cross‐sectional association between KF and risk of cognitive impairment.

Kidney function		Model 1		Model 2		Model 3		Model 4	
	No. of participants	OR (95% CI)	*p*	OR (95% CI)	*p*	OR (95% CI)	*p*	OR (95% CI)	*p*
eGFR quaterfile			**< 0.001**		**< 0.001**		**< 0.001**		**< 0.001**
Q1 (9.30–58.98)	841	1		1		1		1	
Q2 (58.98–74.09)	841	0.788 (0.636–0.976)	**0.029**	1.057 (0.840–1.332)	0.635	1.044 (0.823–1.325)	0.722	1.031 (0.811–1.312)	0.803
Q3 (74.10–85.50)	841	0.601 (0.481–0.751)	**< 0.001**	0.739 (0.584–0.937)	**0.018**	0.693 (0.543–0.885)	**0.005**	0.676 (0.528–0.866)	**0.003**
Q4 (85.50–114.95)	841	0.183 (0.135–0.247)	**< 0.001**	0.542 (0.381–0.770)	**< 0.001**	0.545 (0.380–0.783)	**0.003**	0.530 (0.368–0.763)	**< 0.001**
eGFR Categories			**< 0.001**		0.096		**0.045**		**0.033**
Normal KF	2470	1		1		1		1	
Impaired KF	894	1.988 (1.665–2.373)	**< 0.001**	1.189 (0.978–1.445)	0.083	1.225 (1.001–1.498)	**0.048**	1.251 (1.021–1.534)	**0.031**
Low KF	551	1.749 (1.411–2.167)	**< 0.001**	1.096 (0.868–1.384)	0.442	1.111 (0.873–1.413)	0.392	1.135 (0.890–1.448)	0.307
Poor KF	343	2.411 (1.884–3.085)	**< 0.001**	1.339 (1.026–1.748)	0.062	1.413 (1.075–1.858)	**0.026**	1.441 (1.093–1.900)	**0.020**
ACR quaterfile^a^			**< 0.001**		**< 0.001**		**< 0.001**		**< 0.001**
Q1 (0–2.06)	764	1		1		1		1	
Q2 (2.06–7.40)	763	1.715 (1.265–2.325)	**< 0.001**	1.171 (0.847–1.619)	0.339	1.129 (0.808–1.579)	0.476	1.145 (0.817–1.604)	0.432
Q3 (7.40–24.21)	763	2.201 (1.639–2.958)	**< 0.001**	1.323 (0.965–1.813)	0.123	1.289 (0.929–1.788)	0.194	1.280 (0.920–1.781)	0.215
Q4 (24.27–270.67)	763	3.826 (2.886–5.072)	**< 0.001**	2.101 (1.553–2.841)	**< 0.001**	1.988 (1.454–2.718)	**< 0.001**	2.007 (1.464–2.751)	**< 0.001**
ACR categories^a^			**< 0.001**		**< 0.001**		**< 0.001**		**< 0.001**
A1	944	1		1		1		1	
A2	1461	2.098 (1.639–2.685)	**< 0.001**	1.391 (1.069–1.809)	**0.014**	1.380 (1.049–1.815)	**0.021**	1.377 (1.045–1.814)	**0.023**
A3	648	3.898 (2.981–5.097)	**< 0.001**	2.266 (1.700–3.019)	**< 0.001**	2.241 (1.664–3.019)	**< 0.001**	2.272 (1.683–3.067)	**< 0.001**

*Note:* Significant results appear in bold.

Abbreviations: A1 (1,2,3), albuminuria (1,2,3); ACR, urine albumin‐to‐creatinine ratio; eGFR, estimated glomerular filtration rate; KF, kidney function; Q1 (1,2,3,4), quartile (1,2,3,4).

Model 1: Unadjusted.

Model 2: Adjusted for age, sex, ethnicity, marital status, and education.

Model 3: Adjusted as in Model 2 and additionally adjusted for smoking, drinking status, and regular physical activity.

Model 4: Adjusted as in Model 3 and additionally adjusted for BMI, medical history of hypertension, diabetes, and stroke.

^a^ Of all 3364 participants enrolled in the cross‐sectional analysis, 3053 enrolled participants had available ACR values.

**FIGURE 2:**
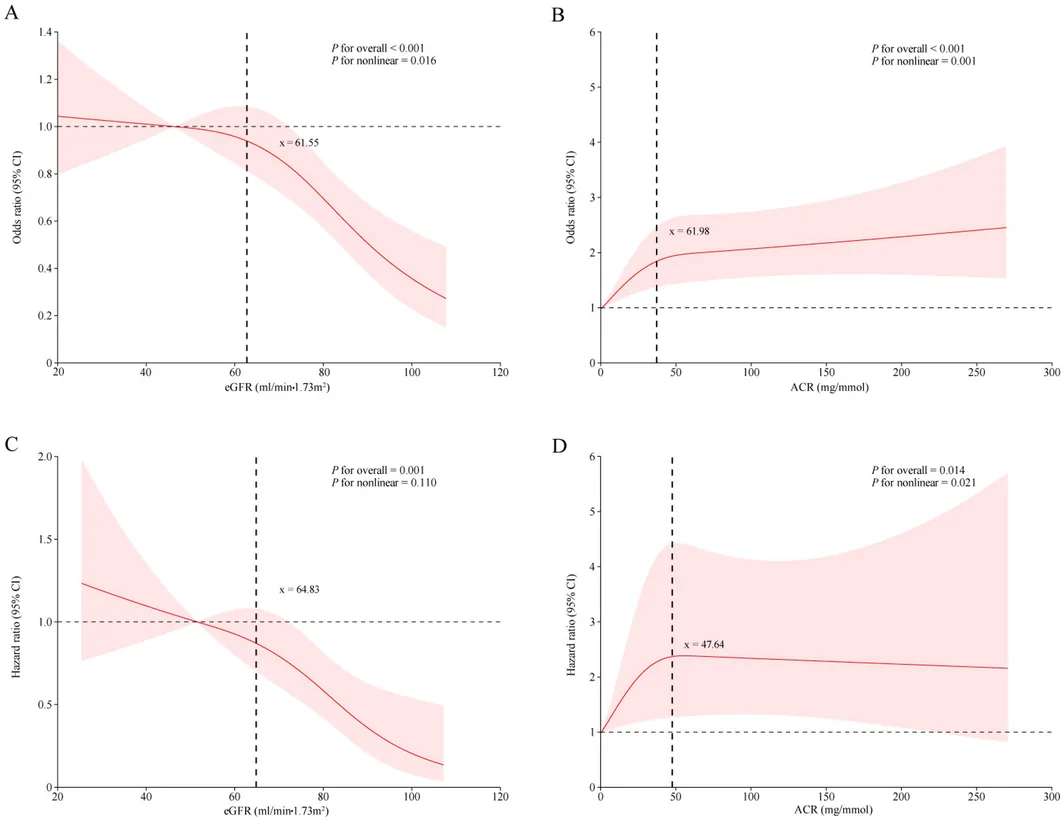
Multivariable adjusted odds ratios (ORs) of prevalent cognitive impairment in relation to eGFR (A) or ACR (B) in the cross‐sectional analysis, and ORs of incident cognitive impairment in relation to eGFR (C) or ACR (D) in the prospective analysis, results from restricted cubic spline regression model. Adjusted for age, sex, ethnicity, marital status, education, smoking, drinking status, regular physical activity, medical history of hypertension, diabetes, and stroke.

### 3.3 Prospective Association Between KF and CI Risk

Among the 1195 participants with normal cognition at baseline in this prospective cohort study, 143 developed cognitive impairment (CI) by 2014. Among participants with impaired KF (eGFR < 60 mL/min/1.73 m^2^; *n* = 244), an estimated 56 developed CI over 3 years (cumulative incidence = 22.95%), compared with approximately 87 cases among those with normal KF (*n* = 951; cumulative incidence = 9.15%). After adjusting for all relevant confounders, participants in the highest two eGFR quartile exhibited a significantly lower odds of incident CI compared to those in the lowest quartile (OR = 0.565, 95% CI: 0.335–0.952 for the third quartile, and OR = 0.299, 95% CI: 0.125–0.715 for the fourth quartile). When eGFR was categorized into normal, impaired, low, and poor KF groups, individuals with diminished KF showed an elevated odds of incident CI relative to those with normal function (impaired KF group: OR = 1.744, 95% CI: 1.162–2.616; poor KF group: OR = 2.090, 95% CI: 1.151–3.797). To assess the robustness of our findings, we conducted a sensitivity analysis using a conventional MMSE cut‐off (< 24) to define cognitive impairment. Under this definition, among participants with normal baseline cognition who had complete follow‐up data (*n* = 1038), 190 incident cognitive impairment cases occurred over the 3‐year follow‐up. Impaired KF (eGFR < 60 mL/min/1.73 m^2^) remained independently associated with an increased risk of cognitive impairment (adjusted OR = 2.211, 95% CI: 1.501–3.257; *p* < 0.001), which was consistent with the primary analysis using MMSE < 18 (adjusted OR = 1.744, 95% CI: 1.162–2.616). These findings suggest that the association between reduced eGFR and cognitive decline is not substantially affected by the choice of MMSE threshold. Regarding ACR, participants in the second, third, and fourth quartiles did not demonstrate significantly different odds of incident CI compared to those in the lowest quartile. However, when ACR was classified according to KDIGO categories (A1–A3), individuals in categories A2 and A3 had a significantly increased likelihood of developing CI compared to those in A1 (A2: OR = 1.801, 95% CI: 1.111–2.920; A3: OR = 2.650, 95% CI: 1.541–4.557) (Table 3). Restricted cubic spline regression revealed a nonlinear relationship between ACR and odds of incident CI in the prospective analysis (*p* for nonlinearity = 0.021). The OR for CI increased sharply with rising ACR up to a threshold of 47.64, beyond which the risk increased more gradually. In contrast, a linear relationship was observed between eGFR and incident CI risk (*p* for nonlinearity > 0.05) (Shown in Figure 2C,D).

**TABLE 3:** Prospective association between KF and incidence of cognitive impairment.

Kidney function	Total number/developed CI	Model 1		Model 2		Model 3		Model 4	
		OR (95% CI)	*p*	OR (95% CI)	*p*	OR (95% CI)	*p*	OR (95% CI)	*p*
eGFR quaterfile			**< 0.001**		**0.019**		**0.024**		**0.022**
Q1 (12.38–63.59)	299/65	1		1		1		1	
Q2 (63.65–78.64)	299/45	0.638 (0.419–0.970)	**0.036**	0.724 (0.466–1.124)	0.150	0.729 (0.468–1.135)	0.161	0.717 (0.459–1.120)	0.143
Q3 (78.74–89.c86)	299/26	0.343 (0.211–0.558)	**< 0.001**	0.562 (0.335–0.943)	**0.044**	0.573 (0.340–0.963)	0.054	0.565 (0.335–0.952)	**0.048**
Q4 (89.86–114.95)	298/7	0.087 (0.039–0.192)	**< 0.001**	0.297 (0.125–0.708)	**0.018**	0.301 (0.126–0.719)	**0.021**	0.299 (0.125–0.715)	**0.021**
eGFR categories			**< 0.001**		**0.017**		**0.021**		**0.019**
Normal KF	951/87	1		1		1		1	
Impaired KF	244/56	2.958 (2.041–4.287)	**< 0.001**	1.760 (1.177–2.632)	**0.006**	1.730 (1.155–2.590)	**0.008**	1.744 (1.162–2.616)	**0.007**
Low KF	167/36	2.729 (1.776–4.194)	**< 0.001**	1.622 (1.019–2.582)	**0.032**	1.589 (0.997–2.532)	0.052	1.591 (0.995–2.543)	0.052
Poor KF	77/20	3.485 (2.000–6.070)	**< 0.001**	2.066 (1.144–3.733)	**0.016**	2.046 (1.130–3.704)	**0.036**	2.090 (1.151–3.797)	**0.030**
ACR quaterfile^a^			**< 0.001**		**0.009**		**0.012**		**0.014**
Q1 (0–0.76)	281/21	1		1		1		1	
Q2 (0.76–3.97)	281/16	0.748 (0.382–1.465)	0.396	0.658 (0.327–1.323)	0.240	0.658 (0.326–1.326)	0.241	0.645 (0.320–1.303)	0.222
Q3 (3.99–13.6)	281/40	2.055 (1.178–3.585)	**0.017**	1.426 (0.786–2.588)	0.243	1.449 (0.797–2.635)	0.241	1.440 (0.791–2.623)	0.233
Q4 (13.8–270.67)	281/56	3.081 (1.810–5.247)	**< 0.001**	1.770 (1.002–3.129)	0.074	1.734 (0.976–3.084)	0.092	1.692 (0.948–3.021)	0.113
ACR categories^a^			**< 0.001**		**< 0.001**		**0.001**		**0.002**
A1	510/32	1		1		1		1	
A2	434/59	2.350 (1.497–3.690)	**< 0.001**	1.765 (1.094–2.849)	**0.020**	1.788 (1.105–2.893)	**0.018**	1.801 (1.111–2.920)	**0.017**
A3	180/42	4.546 (2.765–7.476)	**< 0.001**	2.779 (1.631–4.737)	**< 0.001**	2.712 (1.582–4.652)	**< 0.001**	2.650 (1.541–4.557)	**< 0.001**

*Note:* Significant results appear in bold.

Model 1: Unadjusted.

Model 2: Adjusted for age, sex, ethnicity, marital status, and education.

Model 3: Adjusted as in Model 2 and additionally adjusted for smoking, drinking status, and regular physical activity.

Model 4: Adjusted as in Model 3 and additionally adjusted for BMI, medical history of hypertension, diabetes, and stroke.

^a^ Of all 1195 participants enrolled in the prospective analysis, 1124 enrolled participants had available ACR values.

## 4 Discussion

This large community‐based longitudinal study demonstrates that impaired KF, assessed by both reduced eGFR and elevated ACR, is independently associated with both prevalent and incident cognitive impairment among Chinese older adults.

Although several previous studies have reported that reduced KF is associated with an increased risk of CI [6, 7, 8], others have found no such correlation [13, 14, 15]. These inconsistencies may be attributable to differences in confounding factors, participant characteristics, and methods used to assess KF and cognition. Furthermore, only a limited number of studies have examined the relationship between impaired KF and cognitive performance in older adults [25, 26]. The present study extends these observations to a broader, community‐dwelling elderly population. Notably, by employing a longitudinal design, we found that reduced eGFR and elevated ACR are associated with an increased incidence of cognitive decline, suggesting a temporal relationship that supports a potential causative link. The use of a dual‐parameter, KDIGO 2024–aligned assessment of KF further provides a more comprehensive and clinically relevant evaluation of this association. Collectively, our findings highlight the importance of integrating cognitive screening into the routine care of community‐dwelling older adults with CKD, a recommendation derived from a more generalizable population than previously available.

eGFR based on serum creatinine is widely used in clinical practice to evaluate KF. In the present study, the CKD‐EPI equation was applied to estimate eGFR, as this formula is recommended by the KDIGO 2024 guidelines for being more applicable in the elderly [23]. Evidence indicates that it serves as a good general screening tool for KF, with the advantage of incorporating age and sex into the estimating equation. Additionally, this formula accounts for the complex physiological changes of the kidney and provides a more accurate assessment of renal function in older adults, thereby facilitating disease staging and stratified management [23]. As an effective screening instrument for KF, eGFR benefits from the inclusion of age, race, and sex in its calculation. Although ACR is not yet widely adopted, current European and North American guidelines recommend its use for assessing end‐organ damage in individuals with diabetes, hypertension, and CKD. A meta‐analysis indicated that albuminuria is associated with up to a 35% increased risk of CI [27]. Previous research has demonstrated that albuminuria reflects widespread microvascular endothelial injury in the kidney and an elevated cerebral vascular burden [28]. Endothelial cells are tightly interconnected to prevent hydrophilic substances from entering the brain. Endothelial damage can disrupt the BBB, leading to increased β‐amyloid levels and hyperphosphorylated tau, thereby exacerbating CI and dementia [29]. Furthermore, increased cerebral vascular load may also contribute to CI by aggravating neuroinflammation [28].

Previous investigations have shown that KF is related to total brain volume and medial temporal lobe volume, as well as to higher levels of blood neurofilament light chain (NfL), total tau, p‐tau181, and p‐tau217 in individuals with dementia [5, 9, 10]. Tian et al. highlighted the potential role of the kidney in the physiological clearance of Aβ and tau, supporting the notion that kidney impairment may contribute to AD pathogenesis [4]. One neuropathological mechanism linking impaired KF and dementia may be vascular injury. The kidney and brain share anatomical and functional similarities in microvascular regulation. Juxtamedullary afferent arterioles in the kidney and cerebral perforating arteries are exposed to high pressure, generating a steep pressure gradient over a short distance, which renders them particularly vulnerable to hypertensive damage [30]. Clinically, such hypertensive vascular injury manifests as proteinuria and a progressive decline in GFR in the kidney, and as symptomatic stroke, silent cerebral small vessel disease, and CI in the brain [31]. Another possible mechanism involves inflammatory response and oxidative stress. Previous studies suggest that inflammatory cross‐talk may exist between the brain and kidney [30]. On the one hand, cytokines/chemokines released in CKD can stimulate immune cells, neurons, and glial cells in the brain, initiating a cascade that further produces inflammatory molecules and leads to neuroinflammation and CI. On the other hand, enhanced cytokine interaction may activate the brain renin–angiotensin system (RAS), promoting oxidative stress and dementia [30]. Other potential mechanisms contributing to CI may include the accumulation of uremic toxins due to impaired renal function, BBB disruption‐induced albumin leakage, elevated homocysteine, hypertension, and 25‐hydroxyvitamin D (25‐OH‐VD) deficiency [11, 30, 32].

The strengths of this study include its large sample size and its community‐based longitudinal cohort design. Another advantage is the use of both eGFR and ACR to assess renal function, with classification according to quartiles and KDIGO 2024 criteria. Nevertheless, several limitations should be acknowledged. First, eGFR calculated from serum creatinine is influenced by muscle mass. Since muscle mass tends to decrease in the elderly, this may lead to an overestimation of GFR, potentially obscuring its association with dementia [33]. However, the lack of BMI difference between groups mitigates this concern somewhat. Second, serum creatinine for eGFR calculation was only measured in the 2011 wave of CLHLS and was not available in the 2014 follow‐up wave for our analytical sample. Therefore, we were unable to examine whether dynamic changes in KF (ΔeGFR) predict parallel changes in cognitive function (ΔMMSE) over time. Our prospective analyses are based exclusively on single‐time‐point baseline KF to predict subsequent incident cognitive impairment, rather than evaluating intra‐individual trajectories of renal and cognitive decline. Accordingly, our findings can only establish a baseline risk association and cannot demonstrate direct temporal correspondence between progressive KF loss and progressive cognitive deterioration. Future studies with repeated eGFR and cognitive assessments across multiple waves are needed to clarify the temporal relationship between KF decline and cognitive deterioration. Third, cognitive impairment was defined exclusively by the MMSE score, without distinguishing among specific dementia subtypes such as Alzheimer's disease, vascular dementia, dementia with Lewy bodies, or frontotemporal dementia. The MMSE is a screening tool and cannot capture the full heterogeneity of cognitive disorders or reliably identify underlying etiologies. Of note, kidney dysfunction may contribute to cognitive impairment through divergent mechanisms across dementia phenotypes: impaired peripheral clearance of amyloid‐β and tau may be more relevant for Alzheimer's‐type processes, whereas microvascular injury likely predominates in vascular dementia. Consequently, our observed associations reflect risk for all‐cause cognitive impairment in community‐dwelling older Chinese adults, and we cannot assess differential effect magnitudes across distinct dementia subtypes. Comprehensive neuropsychological assessment and neuroimaging will be necessary for subtype‐specific analyses in future investigations. Fourth, we lacked information on medication use, including antihypertensive agents (e.g., RAS inhibitors, diuretics), antidiabetic drugs, and statins. These medications may influence both KF trajectories and cognitive outcomes through various pathways—for example, RAS inhibitors may slow CKD progression [34], while statins have been variably associated with cognitive protection or impairment [35, 36]. Although we adjusted for the clinical diagnoses that typically prompt these prescriptions (hypertension, diabetes, stroke), we could not account for medication class, dose, duration, or adherence. Diagnosis status serves only as an imperfect proxy for actual drug exposure. Available evidence suggests that residual confounding arising from missing medication data is more likely to bias our effect estimates toward the null, meaning our reported risk magnitudes may be conservative rather than spuriously inflated [37]. Future studies incorporating detailed medication records are needed to validate our findings. Additionally, the study did not include information on the underlying causes of CKD (e.g., diabetic nephropathy, hypertensive nephrosclerosis, glomerulonephritis), as this information was not collected in the CLHLS. The association between kidney dysfunction and cognitive impairment may vary, given that the pathophysiology and vascular burden differ substantially across etiologies. Our overall risk estimates therefore represent an averaged population‐level effect across mixed, uncharacterized CKD causes. Fifth, we lacked data on depressive symptoms and *APOE* genotype—two well‐established risk factors for cognitive impairment. Depression is highly prevalent in individuals with CKD and may independently contribute to cognitive decline via inflammatory and vascular pathways [38, 39]. Although we adjusted for marital status and regular physical activity, these variables cannot fully capture depressive symptomatology. *APOE* ε4 carriage is the strongest genetic risk factor for AD and may modify the cognitive–kidney relationship. Residual confounding by unmeasured depressive symptoms and *APOE* genotype cannot be ruled out and could potentially either inflate or attenuate our risk estimates. It is worth noting, however, that the sex‐modifying effect observed in our study (a stronger eGFR‐cognitive‐impairment association among men) is unlikely to be fully explained by these two unmeasured factors on the basis of existing population‐level evidence. Even so, biological interpretation of this sex interaction requires further validation in cohorts with available neuropsychiatric assessment and *APOE* genotyping.

## 5 Conclusions

This longitudinal study provides robust evidence that kidney dysfunction, characterized by reduced eGFR and elevated albuminuria, is an independent risk factor for cognitive impairment in community‐dwelling older adults. These findings advocate for a more integrated care approach, where cognitive assessment becomes a routine component of geriatric CKD management. Future research should investigate the underlying biological mechanisms and examine whether interventions to preserve KF or reduce albuminuria can delay cognitive decline.

## Author Contributions

X.W. conceived and designed the study, conducted the formal analysis, acquired funding, and reviewed and edited the manuscript. Y.Z. performed the formal statistical analysis, developed the software, and validated the results. Z.W. drafted the original manuscript, contributed to the methodology, and acquired funding. X.L. contributed to the study conception and design, developed the methodology, acquired funding, coordinated and supervised the project, and performed validation. All authors read and approved the final manuscript.

## Funding

This study was supported by the National Natural Science Foundation of China (no. 82401694), the Natural Science Foundation for Young Scientists of Shanxi Province (no. 202303021212339), Science and Technology Project of Guangxi (no. 2024AB33319, 2024JJH140474), Fund Program for the Scientific Activities of Selected Returned Overseas Professionals in Shanxi Province (no. 20230058), and Science and Technology Innovation Program of Higher Education Institutions of Shanxi Province (no. 2023L073).

## Ethics Statement

This study was approved by the ethics committee of Peking University with approval number (IRB00001052‐13074). All study procedures were carried out in accordance with the guidelines of the Declaration of Helsinki.

## Consent

All participants were informed about the purpose of the study, assured of confidentiality, and provided written consent prior to participation. Participation was voluntary, and respondents could withdraw at any time without consequence.

## Conflicts of Interest

The authors declare no conflicts of interest.

## Supporting information


**Figure S1:** Interaction between eGFR and sex on the predicted probability of cognitive impairment. The left panel displays the continuous relationship between eGFR (mL/min/1.73 m^2^) and predicted probability, with shaded areas representing the 95% confidence interval (CI). The right panel shows the predicted probability by eGFR category (≥ 60 vs. < 60 mL/min/1.73 m^2^) with error bars indicating 95% CI. Models were adjusted for age, ethnicity, marital status, education, smoking, drinking status, regular physical activity, BMI, medical history of hypertension, diabetes and stroke. *p* values for interaction were 0.002 (continuous) and 0.024 (categorical), respectively.

## Data Availability

Publicly available datasets were analyzed in this study. This data can be found here: https://opendata.pku.edu.cn/dataverse/CHADS.
